# Impact of COVID-19 on Online Gambling – A General Population Survey During the Pandemic

**DOI:** 10.3389/fpsyg.2020.568543

**Published:** 2020-09-25

**Authors:** Anders Håkansson

**Affiliations:** Department of Clinical Sciences Lund, Psychiatry, Faculty of Medicine, Lund University, Lund, Sweden

**Keywords:** COVID-19, gambling disorder, problem gambling, behavioral addiction, online gambling, sports betting

## Abstract

The COVID-19 pandemic may have severe impact on mental health, and concerns have been raised about potentially increased online behavior and possibly increased gambling problems, such as in sports bettors at risk of transfer to even riskier forms of gambling during sports lock-down. Given the need for objective data about gambling behavior during the pandemic, the present analysis, from a project assessing online gambling in Sweden, aimed to study past-30-day gambling patterns in online gamblers in Sweden. The study, carried out in May, 2020, during the pandemic and its restrictions on society, included past-year online gamblers (*N* = 997). Past-30-day gambling for several gambling types was lower compared to a previous study in online gamblers in the same setting, while online non-sports gambling remained at high levels. Those reporting sports betting even during a period with decreased sports betting occasions proved to have markedly higher gambling problems. COVID-19 may alter gambling behaviors, and online gamblers who maintain or initiate gambling types theoretically reduced by the crisis may represent a group at particular risk.

## Introduction

The COVID-19 pandemic has raised a number of issues related to health, beyond the most urgent and life-threatening consequences. The risk of mental health consequences has been highlighted (Holmes et al., 2020), and this also includes a risk of increased online behavior, such as increased video gaming (King et al., 2020) or addictive internet use (Sun et al., 2020).

Likewise, concerns have been raised about COVID-19-related consequences on gambling behavior. Problem gambling and the gambling disorder represent conditions which are globally widespread; past-year problem gambling has been reported to occur in between 0.1 and 5.8% of the general population across different countries and continents (Calado and Griffiths, 2016). The gambling disorder is associated with severe financial, social and psychological consequences, including psychiatric comorbidity, but can be treated, primarily with cognitive-behavioral therapy and motivational interventions (Potenza et al., 2019).

It has been documented in previous national financial crises that these may affect gambling behavior (Economou et al., 2019), although experiences from such crises are somewhat inconclusive (Olason et al., 2015). The COVID-19 crisis, and the confinement and other restrictions associated with it, represent a previously unseen situation with both financial consequences for the population, other changes to the labor market, schooling and leisure activities. These changes include an increase in the time spent at home, possibly more time spent online, a risk of increased worry about the future, and may potentially affect gambling behavior (Håkansson et al., 2020).

One specific circumstance is the substantial change in the gambling market itself, as virtually all sports typically subject to sports betting disappeared during a significant period of time (Håkansson et al., 2020). Given the possible effects on other types of online behavior from COVID-19-related confinement (King et al., 2020; Sun et al., 2020), it may be hypothesized that some gambling types would be more favored than others, whereas others may decrease (Håkansson et al., 2020). Sports betting is one of types of gambling known to increase the risk of problem gambling, and represents the second most common gambling type reported by treatment-seeking gambling disorder patients in the present setting (Håkansson et al., 2017). One concern is that given the large impact on sports during the pandemic, due to lockdown regulations and canceled events (Radio Sweden, 2020), people with otherwise predominating sports betting habits would turn to gambling types with potentially even higher addictive potential, such as online casino games or other online-based gambling, due to the lack of sports events and land-based gambling opportunities (Håkansson et al., 2020). Early in the pandemic, fear of such a transition within the gambling market led politicians to take legal action, such as through a limit to gambling advertising (SBC News, 2020), or other proposed limits to the extent of online gambling (Reuters, 2020). Sweden, the setting studied here, is one of the countries where the online gambling market is strong and online gambling is common among treatment-seeking patients (Håkansson et al., 2017; Håkansson and Widinghoff, 2020), which contributes to the perceived risk of further online gambling predominance during COVID-19. The fear of a transition to online gambling is supported by the general impression of online gambling being more addictive (Chóliz, 2016).

However, so far, population data are very limited with respect to actual gambling habits during the COVID-19 crisis. A previous general population study (including both gamblers and non-gamblers) in Sweden found a modest percentage reporting an increased gambling behavior during the crisis. As a response to decreased sports events, a minority reported either turning to other remaining sports events, online casino, or horse betting. In total, those reporting an increase in their gambling behavior were more likely to be problem gamblers (Håkansson, 2020). From a population survey in Ontario, Canada, it was reported that during the first weeks of lockdown of physical facilities such as land-based casinos, a substantial migration appeared to have occurred from land-based-only gambling to online gambling (Price, 2020).

Given the theoretically increased role of online behaviors (King et al., 2020; Sun et al., 2020), and the cancelation of sports activities, the present study focused on people reporting to be online gamblers. For the present study, data were derived from a population study on gambling behaviors, designed prior to the COVID-19 crisis as a web survey which was carried out during the month of May, 2020, i.e., during the ongoing crisis. This sub-study used the past-30-day data in order to describe gambling patterns during COVID-19.

The aim of the present study was to describe past-30-day use of different gambling types during the COVID-19 pandemic in individuals defined as online gamblers, in order to enable a comparison with past-30-day data reported from a previous survey in online gamblers carried out in 2018. In particular, given the considerable changes in sports world-wide, the study also aimed to assess whether online sports bettors still reporting past-30-day sports betting differed from those who did not. Here, it was hypothesized that past-30-day gambling in Swedish online gamblers would be more common (in relation to a measure of past-year gambling behavior) for some gambling types, such as online casino or other online-based gambling types likely to be unaffected by the COVID-19 constraints, compared to gambling types more clearly affected by the pandemic. Also, it was hypothesized that people who maintained gambling during the crisis, particularly for gambling types such as sports betting believed to be reduced during COVID-19-related restrictions, would present other characteristics than other online gamblers.

## Methods

### Study Design

The present analysis is a partial analysis from a larger study on online gambling in Sweden. This sub-analysis focuses on past-30-day and past-year gambling patterns in Swedish online gamblers, in order to highlight the online gambling situation during the ongoing COVID-19 crisis. The overall study was designed prior to the COVID-19 crisis, and aimed for a larger number of analyses of the online gambling behavior in the setting. As a past-30-day measure for different gambling types is available, this subset of data was used for the present analysis. The survey was carried out from May 5 to 12, such that the 30-day period for each participant refers to a period well within the period of time when constraints due to COVID-19 were actively ongoing, and during that whole 30-day period, sports betting related to major sports events, such as major soccer leagues, were completely canceled. The study data was collected using the same inclusion criteria and the same methodology as in a previous study carried out in 2018, and which previously has reported associations between specific types of online gambling patterns and problem gambling and indebtedness (Håkansson and Widinghoff, 2020).

The study was reviewed by the Swedish Ethical Review Authority (file number 2020-00364), which expressed that the study did not formally require ethical permission according to Swedish law, as it does not deal with data that can be directly or indirectly linked to a specific individual, and also expressed no ethical concerns with respect to the study. The study was opened only after a participant provided informed consent. Participation in the study was paid following the credit system used by Ipsos for other studies, and where a survey of the present extent and duration is rewarded with credit points corresponding to a value of around 1.50 Euros within the credit system of the company.

### Setting

The present study took place in Sweden, where gambling, since January 1st, 2019, is regulated in a license-based system, with a large number of licensed operators. Land-based casinos and land-based electronic gambling machines are run by a state-based monopoly, whereas betting on sports and horse racing, online casino and bingo games, as well as land-based and online lotteries, are subject to competition between a number of operators. A large percentage of the gambling advertisements seen in television promote online gambling, with online casino representing the largest share of these commercial messages (Håkansson and Widinghoff, 2019). Likewise, a majority of treatment-seeking gambling disorder patients report online casino as their predominating gambling type, with sports betting being the second most common type (Håkansson et al., 2017). Slightly below 1.5% of the general population are believed to be problem gamblers, with an increase reported to have occurred particularly in women, according to official general population survey data (BBC, 2019). During the period analyzed in the present study, sports events on competitive level in Sweden were canceled, whereas land-based horse track racing continued, although without present audience but available through wagering online. Likewise, the four major land-based casinos, all owned by the state monopoly, were closed.

### Participants

The present study aimed to include past-year online gamblers. The sample addressed were web panel members of a Swedish market survey company, Ipsos, i.e., individuals already enrolled with that company’s web panel, and typically receiving market surveys and political opinion polls. The same methods and the same recruitment strategy were used in a previous study assessing online gamblers in Sweden, recruited through the same web survey company and with the same screen-out question (Håkansson and Widinghoff, 2020). Participants of the web panel are regularly addressed with offers to participate in different surveys. In this case, they were included with the question “if you think about the past 12 months, how often have you gambled on sports betting or online casino games?” with the options to respond “don’t gamble on sports betting or online casino,” “1–4 times,” “5–9 times,” “10 times or more,” or “unsure/don’t know.” Only individuals responding “10 times or more” were further considered in the study. The study had the intention to include 1,000 individuals. When closing the study, 1,007 individuals had answered the survey. For 13 of them, at least one of the nine items of the gambling severity instrument (PGSI, see below) were missing, and therefore could not be categorized in a gambling severity category. Three of them, however, had a total value already reaching above the cut-off for the highest problem level in that instrument (eight points or more) from the available items, and were accordingly categorized into that highest problem gambling category and included in the study. The remaining 10 individuals were excluded from further analyses (based on the uncertainty of their problem gambling status), such that a final sample of 997 individuals were included in the study.

### Measures

Patterns of recent gambling was measured for each of the gambling types included, asking for whether that gambling types had been used (1) during the past 30 days, and if not (2) at any time during the past-year (gambling types assessed were online casino, land-based casino, online horse betting, land-based horse betting, sports live betting, sports non-live betting, online poker, land-based poker, land-based electronic gambling machines, online bingo, and gambling within video games). Thus, respondents endorsing the past-30-day item were not asked about the period of time prior to the past 30 days. Individuals reporting any past-year gambling for a gambling type, but not past-30-day gambling for that type, were compared to those reporting past-30-day gambling (non-recent vs. recent gamblers). As no comparable 30-day period was available for comparison, the proportions of past-year gamblers who reported past-30-day gambling, for each gambling type, were used as a measure of the extent to which different gambling types were affected by the COVID-19 period. Problem gambling severity was measured using the Problem Gambling Severity Index (PGSI), a nine-item scale (Wynne and Ferris, 2001) frequently used for the measure of a hazardous or problematic gambling behavior, with questions asked with a time frame of the past 12 months. The same instrument was used, among other studies, in the preceding study on online gamblers in the present setting (Håkansson and Widinghoff, 2020). As in previous research, respondents were categorized as having no risk gambling (0 points), low risk gambling (1–2 points), moderate-risk gambling (3–7 points), or problem gambling (8 points and above). Gender and age (the latter in age groups) were reported, as well as living conditions (categories collapsed into living alone without children vs. not living alone) and occupation (categories collapsed into working/studying vs. unemployed/retired/sick-leave). Also, it was reported whether the individual had ever self-excluded from gambling through the national self-exclusion system *Spelpaus*^1^, a governmental authority-based system introduced in Swedish gambling legislation since January 1st, 2019, and which allows a person to self-exclude for a duration of up to 12 months (with the possibility of prolongation) from all legal (licensed) gambling operators in the country.

### Statistical Methods

Sample characteristics and gambling patterns were reported as descriptive data. Also, for each gambling type, descriptive data report the percentage of past-year gamblers for that gambling type who report having used it during the past 30 days. Past-30-day gamblers—for each gambling type—were compared to non-30-day past-year-gamblers for that gambling type, using chi-square analyses.

## Results

Seventy-five percent of respondents were men, and a majority were either working or retired. In total, 7% had a history of self-exclusion from the *Spelpaus* system. Fifty-two percent had no risk gambling according to the PGSI measure, 23% had low-risk gambling, 15% were moderate-risk gamblers, and 10% were problem gamblers. A full description of the characteristics of the study sample is found in Table 1.

**TABLE 1:** Characteristics of included individuals (*N* = 997).

	n (%)
Male gender	744 (75)
Age groups (years)	
18–24	11 (1)
25–29	45 (5)
30–39	134 (13)
40–49	162 (16)
50–59	265 (27)
60–69	217 (22)
70 and above	163 (16)
Living conditions	
Alone with children	70 (7)
Alone without children	246 (25)
With partner and children	304 (30)
With partner without children	363 (36)
With my parents	14 (1)
Occupation	
Working	600 (60)
Studying	18 (2)
Unemployed	38 (4)
Retired	309 (31)
Other	32 (3)
History of national self-exclusion	
Yes	66 (7)
No	925 (93)
Wish not to answer	6 (1)
Gambling severity	
No risk	514 (52)
Low risk	230 (23)
Moderate risk	154 (15)
Problem gambling	99 (10)

In women (*n* = 253), 17% were moderate-risk gamblers and 20% were problem gamblers (a total of 37%), and in men (*n* = 744), the corresponding percentages were 15 and 6 % (total 21%, *p* < 0.001 for gender difference, chi-square linear-by-linear).

### Patterns of Past-30-Day Gambling

Expressed as the percentage of past-year gamblers who gambled during the past 30 days, for each gambling type, this ratio of past-30-day gambling was the highest for online horse betting (90%), online casino (81%), online poker (74%) and online bingo (72%), as well as for the less frequent gambling within video games (86%), but lower for sports live betting (58%), non-live sports betting (56%), electronic gambling machines (46%), land-based horse gambling (42%), and land-based casino games (26%, Table 2).

**TABLE 2:** Reporting of any past-year gambling (past 30 days or past-year prior to past 30 days), and past-30-day gambling, for all gambling types (*N* = 997).

	Total, any past-year gambling (past-year or past 30 days), percent of all study participants, n (%)	Past-30-day gambling, n	Past-30-day gambling, proportion of all past-year gamblers (past-30-day gambling + other past-year gambling), %
Sports live betting	474 (48)	277	58
Sports non-live betting	495 (50)	279	56
*Total: Any sports betting*	*619 (62)*	*400*	*65*
Online casino	381 (38)	310	81
Land-based casino	81 (8)	21	26
Online horse betting	646 (65)	584	90
Land-based horse betting	291 (29)	123	42
Online poker	178 (18)	131	74
Land-based poker	87 (9)	45	52
Land-based electronic gambling machines	113 (11)	52	46
Online bingo	220 (22)	159	72
Gambling within video games	78 (8)	67	86

For those reporting past-30-day gambling, compared to those denying that but reporting past-year gambling for the same gambling type, being a moderate-risk or problem gamblers was significantly more likely among the recent gamblers for land-based casino gambling, land-based electronic machine gambling, and for any sports betting, but less likely for online horse betting. The past-30-day gamblers for online casino and land-based poker were significantly more likely to be female, whereas the recent online horse bettors were significantly more likely to be men (Table 3). The percentage of respondents in active work or studying were lower in recent gamblers for online casino (68 vs. 86%, *p* < 0.01) and for online horse betting (59 vs. 74%, *p* = 0.02), whereas no significant differences were seen in other gambling types (data not shown).

**TABLE 3:** Comparison of recent (past-30-month) gamblers and past-year (non-recent) gamblers for each gambling type (*N* = 997), chi-square analyses.

	Moderate-risk or problem gambling			Male gender		
	Past-30-day gamblers (%)	Non-30-day (but past-year) gamblers (%)	*p*-value	Past-30-day gamblers (%)	Non-30-day (but past-year) gamblers (%)	*p*-value
Sports live betting	36	22	0.001	83	84	0.76
Sports non-live betting	28	22	0.12	87	83	0.19
*Total: Any sports betting*	*30*	*20*	<*0.01*	*84*	*80*	*0.23*
Online casino	49	41	0.21	55	76	0.001
Land-based casino	81	47	<0.01	57	67	0.43
Online horse betting	23	35	0.03	80	68	0.03
Land-based horse betting	28	24	0.53	78	82	0.46
Online poker	50	38	0.18	73	81	0.26
Land-based poker	58	43	0.16	64	83	<0.05
Land-based electronic gambling machines	69	41	0.003	62	69	0.42
Online bingo	51	46	0.50	53	51	0.73
Gambling within video games	58	73	0.36	63	55	0.61

### Characteristics of Past-30-Day Sports Bettors Compared to Past-Year Sports Bettors

Among respondents reporting any sports betting during the past-year (*n* = 619), those who reported past-30-day sports betting (*n* = 400) were more likely to report past-30-day online casino gambling (30 vs. 22%, *p* < 0.05), land-based casino gambling (5 vs. 0%, *p* = 0.001), online poker gambling (22 vs. 11%, *p* < 0.001), land-based poker gambling (8 vs. 3%, *p* = 0.001), land-based electronic gambling machines (9 vs. 1%, *p* < 0.001), online bingo (18 vs. 12%, *p* < 0.05), and gambling within video games (11 vs. 4%, *p* < 0.01), while there were no significant differences regarding other types of gambling. Those who reported past-30-day sports betting were more likely to have a history of indebtedness (11 vs. 6%, *p* = 0.04), and had higher levels of gambling problems (*p* < 0.001, linear-by-linear, with the proportions of moderate-risk and problem gamblers being 18 and 13% vs. 16 and 5% vs., respectively). Instead, they did not differ with respect to gender, age, history of self-exclusion, living alone without children, or currently in work/studies (data not shown).

## Discussion

The present study is among the first studies reporting recent online gambling data from the COVID-19 crisis. The present study included online gamblers, and focused on the characteristics of those reporting or not reporting recent gambling, in a situation with a changing gambling market where all major sports events had been canceled world-wide. Thereby, the study attempts to shed light onto the discussion about whether the dramatic changes in the society during COVID-19 could affect gambling among online gamblers. In summary, it can be concluded that online gambling types were more common compared to their past-year rates than were the land-based gambling types. Importantly, sports bettors who did report sports betting even during this period, where such betting in the society was assumingly rare, had a very high degree of gambling problems and indebtedness, and gambled more. There was no indication that past-year sports bettors who denied betting in the recent COVID-19-affected period would have an increased gambling on other types of gambling. However, online horse bettors appeared to have a lower degree of gambling problems if they were recent gamblers, such that the characteristics of this group of gamblers may have been different during the pandemic than in the months prior to that.

In the current study carried out during the COVID-19 pandemic, the rates of 30-day gambling in the present study can be compared to the findings of a previous study with the same methods for recruitment, carried out in 2018 (Håkansson and Widinghoff, 2020). In that study, the gender distribution was virtually the same as here (78% men in the previous study), whereas in the present study, participants tended to be older; in the previous study, 4% were in the youngest age group (1% here), and 14 and 8% were in the two oldest age groups (22 and 16% here). In the present study, past-30-day gambling was comparable to the previous study for online casino (31 vs. 34% in the previous study) and online bingo (16% in both studies), whereas gambling types which were lower in the present study include land-based casino gambling (2 vs. 9% in the previous study), land-based horse betting (12 vs. 22%), live sports betting (28 vs. 54% in the previous study) and non-live sports betting (28 vs. 60% in the previous study), land-based electronic gambling machine gambling (5 vs. 10% in the previous study), and online poker (13 vs. 18% in the previous study). Instead, past-30-day gambling in the present study was higher for online horse betting (59 vs. 40% in the previous study). While respondents in the present study tended to be older, the data still describe clearly that land-based gambling types were markedly lower this time, whereas the percentages for online casino and online bingo appeared to be unchanged during the COVID-19 situation. Thus, although movements between gambling types cannot be analyzed here, the present data confirm the hypothesis that during the pandemic, some gambling types are more likely maintained than others, in line with the reported changes to the gambling market during the pandemic, whereas other types are more likely affected. For example, a low reporting of land-based casino gambling was far from surprising, as the major official casinos were closed during the study period, although smaller restaurant-based casinos may still be operating in many places in the country. This is consistent with the description of a relatively substantial migration of gamblers from land-based gambling opportunities to online gambling during casino lockdown in Ontario, Canada (Price, 2020).

The higher degree of gambling problems and indebtedness in past-month gamblers were consistent with the hypothesis that in times where gambling of some types is scarce, those who still engage in that gambling type differ from those who do not. In this context; in times when sports betting is scarce, those who still bet on the reduced amount of sports are likely to have more severe gambling problems. In a recent general population study, a minority of respondents reported that the reduced sports betting opportunities made them gamble on other sports events than they usually do (Håkansson, 2020). In the present study, land-based gambling options, such as casino and gaming machine gambling in the land-based modality, also displayed the same pattern. Thus, even though this was a sample recruited for their online gambling patterns; those who did report recent gambling on the markedly reduced land-based gambling types, had more severe gambling problems. In COVID-19 and potential future similar crises, preventive efforts and interventions should address individuals who maintain gambling behaviors which are abandoned by a majority due to physical and legal restrictions.

Sports betting was far from inexistent even during the weeks when the global restrictions from COVID-19 were the largest, such as during confinement in many countries. Here, it should be borne in mind that individuals were recruited based on their past-year gambling online on 10 occasions or more, i.e., they are likely to be a high-risk sample with respect to online involvement and intense gambling patterns, as supported by a previous study using the same recruitment strategy (Håkansson and Widinghoff, 2020). Thus, the present study may capture a group with particularly pronounced involvement in gambling and low tendencies to give up gambling completely. Also, it is clear that despite the nearly total lock-down of well-established sports world-wide, some sports events still did occur. For example, there have been reports of low-tier soccer games receiving disproportional attention on betting sites, which has been highlighted mainly in the context of fears of fraud (match-fixing). However, besides this type of amateur-level sports events still happening (SBC News, 2020), some nations’ soccer leagues, otherwise unseen in the global media, continued; the Belarus soccer league, for example, received some attention as it remained available for legal sports betting (The Guardian,, 2020). Therefore, again, despite a very large decrease in sports-related gambling opportunities world-wide, individuals who stick to the few gambling options left on the market may be a group presenting particularly high risk of gambling problems.

It has been discussed whether specific other gambling types would attract new users because of the COVID-19, with the fear that some gambling types would put ex-bettors into more addictive gambling because of turning to other than the preferred gambling type. In the present study, for most gambling types, the past-30-day gamblers either did not differ from past-year gamblers, or had a higher degree of gambling problems, such as for sports betting (as discussed above), land-based electronic gambling machines, or land-based casino. It is difficult to know whether the enhanced gambling problems in these recent gamblers are due to a recent increase because of the pandemic, or simply because frequent gamblers are more likely to report recent gambling compared to a person who gambles only occasionally, and therefore likely with a lower degree of problems.

Interestingly, however, one specific gambling type demonstrates the opposite trend; online horse bettors had significantly less gambling problems if they reported past-30-day use, compared to past-year users with no recent use of that type. Although this was measured in a limited sample and can be subject to confounding factors unknown here, it can be hypothesized that this specific gambling type has attracted individuals during the pandemic who have less gambling problems and who typically do not engage in horse race betting, such as if a move had happened from other more pandemic-influenced gambling types to this one. The relatively high reporting of online horse betting (and higher than in our previous study from the same setting) is in line with media reports of a sharp increase in horse wagering during the crisis (Financial Times, 2020), and with the previous reporting from the general population in Sweden that the ratio of individuals increasing/decreasing their horse wagering was unsurprisingly higher than for sports betting which was largely canceled (Håkansson, 2020).

In contrast to the association with gambling problems (and indebtedness), it is interesting to note that employment status, or living alone, were factors unrelated to the reporting of recent sports betting; thus, this study gives no support to the idea that living conditions or a labor situation affected by the crisis may change gambling patterns. However, this issue would require more research, including more detailed and in-depth analyses including longitudinal study designs, and likely would merit from a longer time frame to study than only the weeks of crisis preceding this study.

The present sub-analysis, describing sports betting and other gambling behaviors in online gamblers during a unique change to the society and to the gambling market, may have implications in immediate association with the COVID-19 crisis. For the remainder of the acute and sub-acute phases of the pandemic, those who bet even on a scarce betting market may be more likely to have gambling problems and should be particularly approached by responsible gambling strategies. Likewise, both in the short and long run, the present findings call for more research following gamblers over time during and after the pandemic, and particularly interventions research testing methods to prevent excessive gambling in the context of this crisis. Such interventions may involve legal constraints on gambling types perceived to be particularly hazardous, in particular rapid online games, such as the limitation of advertisements or deposit limits suggested by policy makers in some settings (Reuters, 2020; SBC News, 2020). The actual effect of such interventions remains to be studied. Interventions may also involve an increased awareness in mental health care or social support settings, where hazardous gambling patterns can be screened for in times of a financial crisis. Although the world has never seen a crisis similar to the present one, study implications may also be relevant to other crises of a magnitude affecting many parts of society, including the world of sports and gambling. Also, again, it puts attention to the importance to address the role of gambling in sports; for example, previous research has shown that elite athletes (Grall-Bronnec et al., 2016) may have a higher risk of being problem gamblers.

In the present study, problem gambling was more common in women. While this may be a surprising finding in relation to most previous research, where a majority of problem gamblers are male (Tavares et al., 2001; Díez et al., 2014; Calado and Griffiths, 2016; Edgren et al., 2017). However, in the present setting, female problem gambling may have increased in recent years (Svensson and Romild, 2014; BBC, 2019), and the gender distribution of the whole sample and the sub-sample with moderate-risk or problem gambling is consistent with the previous study using the same methodology (Håkansson and Widinghoff, 2020).

The present study has limitations; it relies on self-report data collected through a market survey company, which may limit the preciseness of reported data. The present study had the intention to include online gamblers, i.e., individuals with a certain degree of online gambling behavior (ten or more occasions during the past-year), given the high prevalence of online gambling and in order to provide a new measure to compare to a previous online gambling study carried out in the present setting (Håkansson and Widinghoff, 2020). Therefore, the present findings cannot be readily generalizable to samples of typical land-based gamblers (but also was not intended to do so), and were studied in only one country (where online gambling is common in problem gamblers, Håkansson et al., 2017), and may not be generalizable to settings where online gamblers represent a smaller proportion of the overall population gamblers. While some key figures were comparable to the previous study in online gamblers in Sweden, the study can only claim to be representative of web panel-recruited online gamblers, and not to represent the whole population of land-based gamblers as well. While lock-down decisions due to the COVID-19 pandemic clearly affected other types of land-based gambling that gambling related to sports, the sample assessed here was included because of their past-year online gambling, making conclusions more difficult to draw conclusions about populations who may have had only a land-based casino gambling, for example.

Likewise, data rely on self-report rather than on objective measures of actual gambling, which, however, would have been difficult given the large number of gambling operators available in the area. It is also not possible to establish, from the present data, whether an individual’s recent gambling represents an initiation or an increase in gambling, or even an individual’s typical pattern of irregular or rare gambling which happened to occur during the past 30 days prior to taking the survey. Related to this, another limitation is the cross-sectional study design, i.e., the lack of a possibility to follow each individual’s changing gambling pattern over time. However, the present analyses aimed to assess the gambling patterns in online gambling during the most acute phases of the pandemic in the present setting, but future follow-up studies are planned using the same type of recruitment, and can provide new measures of how gambling behaviors may alter in post-acute phases of the pandemic. Overall, the results of the present study call for new data collections in this and other geographical settings, and in different pandemic phases. Despite these limitations, the present sub-study from a structured web survey dataset of online gamblers, is one of the first and one of the few studies reporting gambling involvement actually happening during the COVID-19 crisis.

In conclusion, the present study has implications of relevance to stakeholders in the gambling policy area and in preventive and treatment work in problem gambling. People reporting sports betting in times when the world of sports is dramatically altered due to the pandemic may be at higher risk of problem gambling than other sports bettors, and should be a group to address for prevention and intervention. Online casino and bingo gambling appear to less affected by the COVID-19 crisis, while land-based gambling in these online gamblers appeared to be more scarce, and online horse betting was the only gambling type more commonly reported than in a corresponding previous dataset. The present findings add to the knowledge about online gambling, and to the need to address online gambling as one of the potential health hazards in the aftermath of the COVID-19 pandemic.

## Data Availability Statement

The raw data supporting the conclusions of this article will be made available by the authors, without undue reservation, to any qualified researcher.

## Ethics Statement

The studies involving human participants were reviewed and approved by the Swedish Ethical Review Authority. The patients/participants provided their written informed consent to participate in this study.

## Author Contributions

AH was the sole author of the present manuscript, and the responsible of the research idea, the planning and ethics application of the study, as well as statistical analyses and writing of the manuscript.

## Conflict of Interest

AH holds a position as researcher at Lund University which was sponsored by the Swedish state-owned gambling operator Svenska Spel as part of that body’s responsible gambling policies, and also had funding from the research council of Svenska Spel as well as the research council of the Swedish alcohol monopoly and from the Swedish Sports Federation. None of these organization have been involved in or had any influence on any part of the present work.

## Footnotes

1 spelpaus.se
